# Adenosine Receptor Antagonists to Combat Cancer and to Boost Anti-Cancer Chemotherapy and Immunotherapy

**DOI:** 10.3390/cells10112831

**Published:** 2021-10-21

**Authors:** Rafael Franco, Rafael Rivas-Santisteban, Gemma Navarro, Irene Reyes-Resina

**Affiliations:** 1 CiberNed, Network Research Center, Neurodegenerative Diseases, Spanish National Health Institute Carlos III, 28034 Madrid, Spain; rrivasbioq@gmail.com (R.R.-S.); g.navarro@ub.edu (G.N.); ire-reyes@hotmail.com (I.R.-R.); 2 Department of Biochemistry and Molecular Biomedicine, University of Barcelona, 08028 Barcelona, Spain; 3 Department of Biochemistry and Physiology, Faculty of Pharmacy and Food Science, University of Barcelona, 08028 Barcelona, Spain

**Keywords:** A_2A_ adenosine receptor, A_2B_ adenosine receptor, clinical trial, carcinoma, metastases, chemoradiation, glioma, neuroblastoma

## Abstract

Extracellular adenosine accumulates in the environment of numerous tumors. For years, this fact has fueled preclinical research to determine whether adenosine receptors (ARs) could be the target to fight cancer. The four ARs discovered so far, A_1_, A_2A_, A_2B_ and A_3_, belong to the class A family of G protein-coupled receptors (GPCRs) and all four have been involved in one way or another in regulating tumor progression. Prompted by the successful anti-cancer immunotherapy, the focus was placed on the ARs more involved in regulation of immune cell differentiation and activation and that are able to establish molecular and functional interactions. This review focuses on the potential of A_2A_ and A_2B_ receptor antagonists in cancer control and in boosting anti-cancer chemotherapy and immunotherapy. The article also overviews the ongoing clinical trials in which A_2A_R and A_2B_R ligands are being tested in anti-cancer therapy.

## 1. Introduction

Adenosine (Ado) was considered a molecule that protects against cancer. One example is muscle, which is always involved in ATP/Ado/ATP cycles. In fact, striated muscles have little risk of metastasis implantation. Twenty years ago, it was discovered that oral administration of a muscle cell conditioned medium inhibited tumor growth in mice [1]. Adenosine was responsible for the effect and the action was mainly mediated by the A_3_ receptor, which is one of the four identified proteins that sense extracellular adenosine: A_1_, A_2A_, A_2B_ and A_3_ [2] (see below for a general description of adenosine receptors). Interestingly, it was postulated that, in addition to adenosine, muscle cells produced other compounds (“stable, nondegradable”) that behave as agonists of the A_3_ receptor (A_3_R) [1].

It has been assumed that extracellular adenosine plays a key role in minimizing the occurrence of tumors in muscle (heart included). The compound has been defined as “*multi-signaling guardian angel in human diseases*” [3]. However Ado accumulates in the environment of several tumor types and Jozef Spychala wrote in 2000 an article entitled: “*Tumor-promoting functions of adenosine*” [4]. Is Ado friend or foe? As is often the case, the answer is: it depends. From a therapeutic point of view, it is evident that in cancers in which Ado acts as a foe, the use of antagonists of ARs may help, and this is the basis of trials to assess the efficacy of AR antagonists as boosters of chemotherapeutic agents, with potential also in radiotherapy. Due to the fact that ARs mediate the regulation of the immune system by Ado, ligands of those receptors may act as boosters in the relatively recent and highly efficacious monoclonal antibody-based immunotherapeutic anti-cancer approach. Here, we present an overview of the possibility of targeting adenosine receptors (ARs) in the fight against cancer. Due to the fact that the aim of the article is not reviewing all possibilities of all ARs on all cancers, we will focus on the potential of those that have, as of today, more chances to be targeted if Ado-related medication arrives to the bedside of the patient with cancer, namely the A_2A_ receptor (A_2A_R) and the A_2B_ receptor (A_2B_R). Seemingly, there is not any clinical trial registered in ClinicalTrials.gov (CTg) to target the A_1_R in cancer. As for the A_3_R, there are two clinical trials registered to test agonist in hepatic carcinoma; the last study was posted in May 2014 (CTg Identifier: NCT02128958) and results, recently reported, show a good safety profile and modest efficacy [5]. For readers interested in anti-cancer approaches taking adenosine as reference, but from a metabolic perspective, the review by Boison and Yegutkin (2019) is strongly recommended [6]. Authors point out “*the need for a more careful evaluation of the entire purinome in emerging cancer therapies*” and list the clinical trials on the efficacy of anti-cancer therapies of molecules altering the metabolic balance of adenine nucleotides and nucleosides.

## 2. Adenosine and Adenosine Receptors

Ado, the adenine purine nucleoside, is an autacoid, which is defined by Merriam Webster dictionary as: “*a physiologically active substance (such as serotonin, bradykinin, or angiotensin) that is produced by the body and typically has a localized effect of brief duration*”. Ado is found in every cell in the human body. Ado is metabolically related to adenosine triphosphate (ATP) and is therefore present in almost any living cell on the Earth’s surface. Apart from its relevance for energy metabolism within the cell, it can be released into the extracellular environment where it becomes an important modulator that acts through specific receptors located on the cell surface. Purinergic P1 receptors, more commonly known as Ado receptors (ARs), belong to class A rhodopsin-like G protein-coupled receptors (GPCRs) and four have been discovered so far. Before the cloning technique was available, pharmacological tools allowed the discovery of 3 receptors that were named as A_1_, A_2_ and A_3_; one more receptor was identified by cloning its cDNA, and the nomenclature that holds today is: A_1_, A_2A_, A_2B_ and A_3_. Whereas A_1_ and A_3_ receptors couple to G_i/o_, whose engagement leads to reduction in the activity of the adenylyl cyclase, the canonical proteins that couple to A_2A_ or to A_2B_ receptors are of the G_s_ family, whose engagement leads to an increase in the activity of the adenylyl cyclase [2]. A comprehensive review on adenosine receptors and cancer was written in 2009 by Fishman et al. [7]. A more recent review provides hints on how Ado receptors have become targets to combat cancer [8]. 

## 3. Therapeutic Drugs Acting on ARs

Despite the huge amount of research on ARs and the potential of ligands acting on them, specially of antagonists, few AR-related drugs have been approved for human use. The first was Ado itself, which can be used in the emergency room to convert life-threatening paroxysmal tachycardia into sinus rhythm. Indeed, the physiological actions of adenosine on the heart were already discovered in the nineteen-twenties by Drury and Szent-Györgyi [9]. A couple of decades later, the laboratory of Berne provided the molecular and clinical basis for the use of Ado in paroxysmal tachycardia [10,11]. Despite the usefulness of Ado in such extreme condition, AR agonists do have, generally speaking, unwanted side effects.

Unlike agonists, AR antagonists are generally safe, and this property has helped in the approval of AR antagonists in two phases. First, the non-selective antagonists, that were already being taken as part of coffee/tea/cola beverages, were approved. Several years later, a selective A_2A_R antagonist was approved as the first drug in its class, opening the door for further approval of AR antagonists in the future. In fact, non-selective AR antagonists, known as methylxanthines due to their chemical structure, were approved several years ago for some conditions such as asthma. The best known methylxanthines are caffeine and theophylline, that have been consumed for centuries in different cultures as components of beverages, such as coffee, tea and mate. Actually, caffeine is the most consumed psychoactive substance in the world (present in coffee, tea and cola drinks). Importantly, methylxanthines as the recently approved AR-related drug: istradefylline are safe. Istradefylline is a selective antagonist of the A_2A_R that was first approved in Japan (Nouriast^®^) and later in the US (Nourianz^®^) as adjuvant in the therapy of Parkinson’s disease [12,13,14,15,16]. Regadenoson (Lexiscan^®^), an A_2A_R agonist, is not used in therapy but in diagnosis of alterations of myocardial perfusion [17,18,19]. Unfortunately, Regadenoson has been tested, without success, for making the blood–brain barrier more permeable to drugs that need to enter the brain to attack gliomas and neuroblastomas, some of which are very aggressive and have a poor prognosis [20,21].

Ado, like almost all regulatory molecules that act on cell surface GPCRs, exerts multiple actions based on various parameters. Ado action depends on the tissue, it may even be different in different parts of the tissue. Importantly, the expression of receptors that mediate Ado actions may vary in response to various stimuli or may be under circadian variations. One of the best known examples is the regulation of AR expression in response to caffeine intake [22,23]. Taking the kidney as a model, the expression of the four ARs happens to be heterogeneous when considering the different anatomical regions [24,25,26,27]; even in a single renal tubular epithelial cell, the expression may be fairly different in the basolateral and luminal membranes [28,29]. Finally, a given AR may be present in different cell types in different anatomical regions of a given tissue, the most extreme case being the brain, where ARs are heterogeneously distributed with eventual enrichments such as the high expression of the A_2A_R in the striatum. On the other hand, AR in tumor cells may be up or down regulated and each AR type may have opposite roles, thus raising doubts on the best way to attack a given cancer. A very detailed review on the expression of ARs in different tumor cells lines and the signaling pathways and effects, among them proliferative versus antiproliferative, following AR activation has been recently provided by Kazemi et al. [30]. In a recent review on breast cancer, it is stated that “*among the P1 receptors, the A_1_, A_2A_, and A_2B_ receptors are involved in the proliferation and invasion of breast cancer, while the A_3_ receptor is related to the inhibition of tumor growth*” [31]. In this complex scenario, is targeting ARs a therapeutic option in anti-cancer therapy?

At the preclinical level, there have been several investigations aimed at combating cancer using ligands targeting ARs. As many of the approved drugs targeting GPCRs, those ligands are designed and prepared for oral administration. However, it is quite difficult to find a certain cancer that can be cured by targeting ARs. The main role of AR activation is not the suppression or induction of gene expression, and consequently, it is difficult to find a scenario in which tumor growth can be stopped by targeting these receptors. Despite these theoretical limitations, a compound from Palobiofarma, PBF-1129, which is a selective A_2B_R antagonist, is in phase 1 clinical trials to be further tested for efficacy in locally advanced or metastatic lung cancer (CTg Identifier: NCT03274479). Another selective A_2B_R antagonist, TT-4 (from Tarus Therapeutics), is also being tested in phase1/2 in patients with advanced solid tumors (CTg Identifier: NCT04976660).

It should be noted that there is a dual A_2A_/A_2B_ receptor antagonist, etrumadenant (or AB928), which when tested in phase 1 clinical trials showed good safety scores and excellent pharmacokinetics after oral administration [32]. According to the NCT03720678 trial (ClinicalTrials.gov, now in phase 1/1b), AB928 will be tested for efficacy in gastroesophageal and colorectal cancers; there are also expectations that the compound could be an efficacious adjuvant in radiotherapy, chemotherapy and immunotherapy approaches. Actually, the most likely scenario is that AR activation or blockade could serve as adjunctive therapy in chemotherapeutic and immunotherapeutic (even chemoradiation/radiotherapeutic) approaches to combat a wide variety of cancers. The next two sections aim at answering these questions: (i) is AR targeting an effective means to enhance anti-cancer immunotherapy? and (ii) is AR targeting an effective means to enhance anti-cancer chemotherapy?

Due to the approval of A_2A_R antagonists in the therapy of Parkinson’s disease, the safety of other A_2A_R antagonists has been tested, and they turned out to be generally safe. One of them, which was intended to be in line for Parkinson’s disease, has entered into clinical trials for cancer, even as a monotherapy: NIR178 (PBF-509) in advanced cases of lung cancer (CTg Identifier: NCT02403193; phase 1-2) [33] or TT-10 to treat patients with “*advanced*” solid tumors (CTg Identifier: NCT04969315). The list of ongoing clinical trials targeting A_2A_ or A_2B_ receptors is in Table 1 (source Clinicaltrials.gov; accessed on 7 October 2021).

## 4. Adenosine and Adenosine Deaminase in the Cells of the Immune System 

Adenosine is a key regulatory molecule in the immune system. Adenosine deaminase 1 (ADA1), the enzyme that converts adenosine into inosine, is fundamental for the development of the immune system. In fact, the congenital defect of adenosine deaminase 1 leads to severe combined immunodeficiency (SCID) with lack of T and B cells in the blood of patients. A specific toxicity for lymphoid organs due to accumulation of deoxy ATP was first assumed as responsible of such rare and severe disease. Although gene therapy was attempted, a better option for SCID patients was the replacement therapy using preparations of ADA1 conjugated to polyethylene glycol [34,35,36,37,38,39,40,41,42,43,44,45]. The optimal treatment, however, is the transplantation of bone marrow from a close relative with very similar composition of HLA human leukocyte antigens [46]. 

**Figure 1:**
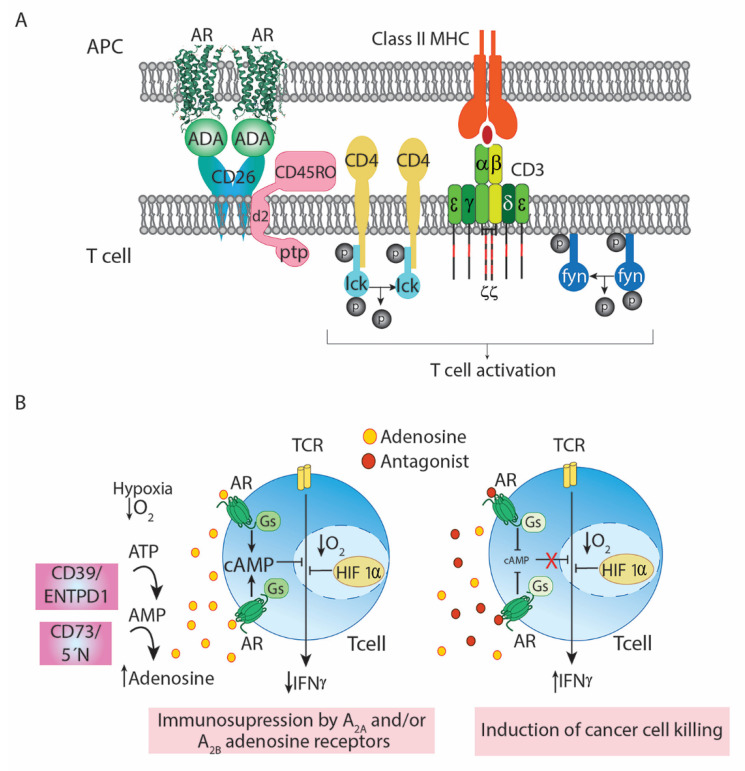
Immunosuppressive actions of Ado in tumor microenvironment and rescue by AR antagonists. Panel (**A**) Proposed model of the CD26/Ado deaminase/AR-mediated co-stimulation of T-cells upon antigen presentation. Protein Src kinases such as Lck (which constitutively binds to the cytosolic domain of T-cell coreceptors CD4 or CD8) and Fyn are activated following TCR/CD3/CD4 cross-linking on T-cell surface promoted by the interaction of Major Histocompatibility Complex (MHC) proteins with antigens in presenting cells. These Src kinases phosphorylate immunoreceptor tyrosine-based activation motif (ITAM) in the cytosolic region of CD3γ, CD3δ, CD3ε, and CD3ζ (red lines). Phosphorylation allows recruitment of other protein tyrosine kinases to the ITAM on CD3ζ, which is phosphorylated and activated by Lck. These other kinases will activate intracellular signaling pathways leading to T-cell activation. In parallel, ADA1 adenosine deaminase anchored to the A_2B_ AR on the antigen-presenting cell surface bridges the T-cell via cell surface CD26, at the same time that antigen is sensed by the T-cell receptor/CD3 complex. Subsequent to T-cell activation, CD45RO phosphatase bound to CD2, (via the d2 intracellular domain) is activated and recruited to the lipid rafts. The protein Tyr phosphatase (ptp) domain of CD45RO catalyzes the dephosphorylation of the regulatory domain of Src kinases including Lck and Fyn, thus inducing Src kinase activation. The model shown is similar for CD4^+^ T cells and CD8^+^ T cells. Abbreviations: d2, intracellular domain d2 of CD45RO; ptp, tyrosine-phosphatase domain of CD45RO. Adapted with permission from ref. [48]. 2007 Copyright Begell House Inc. Panel (**B**) Mechanism by which antagonists of G_s_-coupled ARs rescue the anti-tumor activity of effector T cells. Elevated extracellular (Ado) in the tumor environment leads to immunosuppression. The hypoxia-induced increase in the expression of two ecto-nucleotidases, CD39 (ecto-nucleotide tri(di)phosphohydrolase-1 -ENTPD1-) and CD73 (5′-nucleotidase -5′N-)) in the tumor microenvironment results in enhanced conversion of extracellular adenine nucleotides into extracellular Ado. Activation of ARs leads to increases in cAMP, thus leading to impaired function of the effector cells of the immune system. Hypoxia also stabilizes the transcription factor HIF-1a, which cooperates with the ARs to suppress T-cell effector functions (left). Blockade of G_s_-coupled ARs (A_2A_R and A_2B_R) restores the levels of intracellular cAMP, rescues the immunosuppression and enhances cancer cell killing by effector cells (right). Adapted from ref. [63].

Relevant to the present discussion is the fact that the enzyme, when present in the extracellular or pericellular space, exerts multiple actions. On the one hand, it degrades extracellular adenosine, thus adjusting the level of the natural ARs agonist, whose affinity depends on the subtype. For instance, whereas the Ado/A_1_R binding is considered of high affinity, the A_2B_R displays a very low affinity for Ado. Although poorly studied within the immune regulation context, inosine is considered a low-affinity regulatory molecule that acts on A_3_Rs [1,47]. ADA1 may also act as a costimulatory molecule. This enzyme-independent role is relevant, among others, for lymphocyte differentiation, antigen presentation and lymphocyte activation. We found that ADA1 acts as a bridge between antigen-presenting cells and lymphocytes at the so-called immune synapse (Figure 1A). In this case, the “receptors” for ADA1 are CD26 (also known as dipeptidyl peptidase IV) on one of the cells and ARs on the other cell. At first, it was noticeable the identification of interaction of ADA1 with an AR, but indeed the enzyme may interact with more than one AR type. It is now known that the functionality of ARs is regulated by the enzymatic and extra-enzymatic action of ADA1, and that this is of special relevance in the immune system [48,49,50,51,52,53,54,55,56,57,58]. May inhibition of ADA1 be useful to combat cancer when Ado is friend? Deoxycoformycin (YK-176), a potent and irreversible inhibitor of ADA1, was proven efficacious in hairy cell leukemia as reported in 1992 by Shimoyama and colleagues [59]. Currently, there are several ongoing clinical trials to test efficacy in a variety of cancers of combined treatments that include deoxycoformycin (see https://clinicaltrials.gov/ accessed on 7 October 2021). May supplementation of ADA1 be useful to combat cancer when Ado is foe? This would be easy to test, as pegylated-ADA is already approved for human use. Remarkably, inhibitors of ADA1 may affect the interaction and achieve removal of ADA1, thus increasing local Ado levels and/or allosterically altering ligand binding to AR and AR-mediated signaling [60,61,62].

## 5. A_2A_R Antagonists to Boost Anti-Cancer Immunotherapy

Adenosine and all the proteins that handle or interact with the compound, both the enzymes and the receptors, are relevant to the various sides of the immune system’s action. Let us focus on AR potential as targets to improve the efficacy of anti-cancer immunotherapy. ARs are expressed in every cell of the immune system. However, the receptor type and the expression level vary from cell to cell, and they also vary according to the cell status, for instance, resting versus activated. For a detailed and relatively recent review of the expression of ARs in different immune cells see Vigano et al., 2019 [64].

The work in Michail Sitkovsky’s lab has complemented previous research that shows that the A_2A_R mediates many of the functions of Ado in the immune system [63,65,66,67,68,69,70,71]. Other laboratories have confirmed the findings using novel AR antagonists [72]. The investigation has been intense and prolonged in time but the conclusions can be presented succinctly. The tumor microenvironment has a high concentration of Ado that inhibits the activation of lymphocytes that are capable of killing tumor cells (Figure 1B). The mechanism is based on the increase in cAMP after the activation of G_s_-coupled A_2A_Rs. Consequently, a selective A_2A_R antagonist releases this brake and reduces cAMP levels which, in turn, make lymphocytes capable of effectively fighting/destroying tumor cells (Figure 1B). 

There is a significant number of clinical trials testing the combination of A_2A_Rs with anti-cancer immunotherapies and, eventually, adding inhibitors of the enzyme that converts AMP to adenosine (CD73/5′-nucleotidase, see Figure 1B). A phase 1/1b study has been published examining in patients with “*advanced malignancies*” a selective A_2A_R antagonist, NIR178 (PBF509), an antibody, PDR001, that binds to programmed death-1 (PD-1) protein to restore the anti-tumor activity of effector T lymphocytes and/or a humanized antibody against the 5′-nucleotidase, NZV930 (CTg Identifier: NCT03549000) [73]. A phase 2 trial was posted for the potential of combining NIR178 and PDR001 anti-PD-1 protein antibody in solid tumors and non-Hodgkin lymphoma (CT Identifier: NCT03207867). The primary outcome of this assay is the measurement, in 376 participants, of the response rate every 8 weeks, for 40 weeks. NIR178 is tested as monotherapy (see above) or in combination with as many as 3 different therapeutic agents: DFF332, an inhibitor of hypoxia-inducible factor (Hif)-2α, RAD001, an inhibitor of mammalian target of rapamycin (mTOR), and PDR001 anti-PD-1 protein antibody (CTg Identifier: NCT04895748).

A potential to boost anti-cancer immunotherapy has been attributed to antagonists of the A_2B_R, although with much less preclinical information than that available for A_2A_R antagonists. The underlying mechanism is likely similar since A_2B_R is coupled to G_s_ and, therefore, its activation by Ado leads to increases in cAMP; accordingly, A_2B_R antagonists can reduce cAMP levels and lead lymphocytes to restore their antitumor activities (Figure 1B). Furthermore, there is an interesting interplay between A_2A_ and A_2B_ receptors. Moriyama and Sitkovsky demonstrated that the A_2A_R is involved in A_2B_R expression [74] whereas, more recently, it has been shown that the functionality of A_2A_R is affected by the expression of A_2B_R and that the two proteins may interact to form novel functional units [75,76]. The ultimate significance of these findings is unknown, but the field has evolved to view blockade of these receptors as an effective means of fighting cancer. In fact, there are several clinical trials (in early stages) in which antagonists selective for the A_2A_R, for the A_2B_R or dual (A_2A_R/A_2B_R) are being/will be tested in patients with different cancers.

With the above-described data as background, several clinical trials to assay A_2A_R and/or A_2B_R antagonists as boosters of cancer immunotherapy have been posted. A phase 2 clinical trial, proposed for combating non-small cell lung cancer, uses etrumadenant in combination with two monoclonal antibodies: AB122 (zimberelimab, a monoclonal antibody that binds programmed death-1 protein) and AB154 (domvanalimab; a humanized monoclonal antibody directed against T-cell Ig and ITIM (TIGIT) T-cell domain) (CTg Identifier: NCT04262856). Both PD-1 and TIGIT are key mediators of the fate of tumor antigen-specific effector T cells [77,78]. A similar design (phase 1b/2) to combat “*metastatic castrate resistant prostate cancer*” has been posted using etrumadenant, AB122 and/or an inhibitor of the enzyme producing adenosine from AMP (CD73/5′-nucleotidase) (CTg Identifier: NCT04381832). One of the treatment arms of this trial will use a combination of the dual A_2A_R/A_2B_R antagonist, the AB122 antibody, and standard chemotherapy (enzalutamide or docetaxel). A very similar trial was posted in 2018 and was recently updated, in August 2021, for the therapy of “*advanced malignancies*” (CTg Identifier: NCT03629756). For prostate cancer, another selective A_2A_R antagonist, AZD4635, is being used in phase 2 open-label modular study in combination with oleclumab (MEDI9447), which targets CD73/5′-nucleotidase, with durvalumab (MEDI4736), which targets PD-1 protein, or with both antibodies (CTg Identifier: NCT04089553). 

For refractory of relapsed myelomas, another selective antagonist of the receptor, ciforadenant, is being tested (phase 1) in combination with daratumumab, a monoclonal antibody targeting an activator marker of lymphoid cells, CD38, with multiple enzyme activities that involve adenine nucleotides (CTg Identifier: NCT04280328). The same antagonist is planned to be tested in combination with atezolizumab (Tecentriq^®^), a monoclonal antibody that binds to PD-1, in “*advanced*” cancers (CT Identifier: NCT02655822, phase 1/1b). 

## 6. AR Ligands in Chemotherapeutic Approaches

There are clinical trials to evaluate the efficacy of using AR ligands to enhance cancer therapies. Hopes for the efficacy of adenosine receptor ligands in cancer therapy are greatly increased when chemotherapy enhancers are considered. Although the ligands of the four ARs may be candidates, the two currently best positioned are A_2A_R and A_2B_R antagonists. A dual A_2A_/A_2B_ receptor antagonist is being used in several clinical trials for its potential in different malignancies. One example is the phase 1/1b study to test the possibility of using a combination of etrumadenant and a “classical” chemotherapeutic agent, doxorubicin, in gynecological malignancies or triple negative breast cancer (CTg Identifier: NCT03719326). A more complex study for the same application uses etrumadenant in combination with chemotherapeutic agents, pegylated liposomal doxorubicin or nanoparticle albumin-bound paclitaxel and IPI-549, an inhibitor of is a phosphoinositide-3-kinase-gamma (CTg Identifier: NCT03719326).

Some of the trials that use monoclonal antibodies as the main component of the anti-cancer immunotherapy approach are also assaying A_2A_R and/or A_2B_R antagonists with current chemotherapies. An example is the recently updated phase 1/1b trial to *“evaluate in participants with non-squamous non-small cell lung cancer the safety, tolerability, pharmacokinetic, pharmacodynamic, and clinical activity of etrumadenant in combination with carboplatin and pemetrexed”.* The trial considers to assay this combination of drugs with and without using anti-PD-1 antibodies (pembrolizumab or zimberelimab; CTg Identifier: NCT03846310). 

The dual AR antagonist, etrumadenant, is being tested in phase 1/1b trial in combination with standard chemotherapy consisting of oxaliplatin, leucovorin and 5-fluorouracil, i.e., within a standard mFOLFOX chemotherapy regimen (CTg Identifier: NCT03720678) and also with a mFOLFOX chemotherapy regimen plus zimberelimab plus/minus bevacizumab, an antiangiogenic agent monoclonal antibody (CTg Identifier: NCT04660812). The same study also contemplates an arm in which etrumadenant is combined with zimberelimab and an inhibitor of CD73/5′-nucleotidase. This multicenter randomized phase 1b/2 trial aimed at treating patients with metastatic colorectal cancer is very ambitious and the results may give key information for the design of successful combination therapies.

Finally, it should be noted a phase 1 study that combines the dual AB928 AR A_2A_R/A_2B_R antagonist, is in a phase 1b clinical trial aimed at testing whether the combination with anti-PD-1 (AB122) zimberlimab, a chemotherapeutic agent, cisplatin, and radiotherapy is, in combating locally advanced head and neck cancers, more effective than radiation therapy, single-drug chemotherapy, or other combination drug therapies (CTg Identifier: NCT04892875).

As of today, it is too soon to know whether the different AR antagonists that are being tested will have significant positive effect on boosting chemotherapy beneficial effects. It is, however, likely that more trials will be posted to test, in different cancer types and stages, the interventions in which treatments (chemotherapeutic and/or radiotherapeutic) incorporate A_2A_ or A_2B_ receptor antagonists (or dual A_2A_/A_2B_ receptor antagonists). 

## 7. Conclusions

Adenosine receptor antagonists, which are generally safe, have the potential to boost chemotherapeutic or immunotherapeutic anti-cancer approaches. Due to its relevance in activated immune cells, the A_2A_R is the main target in the immunotherapeutic approach. The A_2B_R seems more promising as a target in chemotherapeutic interventions (even in radiotherapy). The existence of dual compounds, i.e., molecules acting on the A_2A_R and on the A_2B_R, has prompted their use in both immunotherapeutic and chemotherapeutic interventions. Several ongoing clinical trials will, hopefully soon, provide the bases to approve AR ligands in anti-cancer therapy.

## Figures and Tables

**Table 1:** Ongoing clinical trials registered at Clinicaltrials.gov in which A_2A_ or A_2B_ receptors are targeted to combat malignancies.

Target *	+/− Combination Therapy	Company /Institution	Cancer Type	Clinicaltrials.Gov Identifier	Study Phase
A_2A_R	Inupadenant + EOS-448 (anti-TIGIT mAB)	iTeos Therapeutics	AST	NCT05060432	1, 2 (NR)
A_2A_R	Ciforadenant +/− atezolizumab (mAb against PD-1)	Corvus Pharmaceuticals, Inc.	Prostate (advanced/incurable)	NCT02655822	1, 1b (R, OL)
A_2A_R	PBF-509 +/− antibody against PD-1	Palobiofarma SL	NSLC (advanced)	NCT02403193	1, 2 (NR, OL)
A_2A_R	Taminadenant + Spartalizumab (mAb against PD-1) + DFF332 (Hif2α inhibitor)	Novartis Pharmaceuticals	Renal (advanced)	NCT04895748	1, 1b (NR, OL)
A_2A_R	NIR178 +/− Spartalizumab (mAb against PD-1	Novartis Pharmaceuticals	Solid tumor, NHL	NCT03207867	2 (NR, OL)
A_2A_R	PBF -509	Palobiofarma SL	NA (safety assessment)	NCT01691924	1 (R)
A_2A_R	PBF -509	Fundació Institut de Recerca de l’Hospital de la Santa Creu i Sant Pau	Cancer (general)	NCT02111330	1 (R, DB, PC)
A_2A_R	Ciforadenant + daratumumab (mAb against CD38)	Corvus Pharmaceuticals, Inc.	Multiple Myeloma	NCT04280328	1 (OL)
A_2A_R	TT-10	Tarus Therapeutics	Renal, prostate, NSCLC (AST)	NCT04969315	1, 2 (OL)
A_2B_R	TT-4	Tarus Therapeutics	Gastrointestinal, hepatocellular, prostate	NCT04976660	1, 2 (OL)
A_2A_R/A_2B_R	Arm 1: AB928/Etrumadenant + zimberelimab (mAb against PD-1) + enzalutamide Arm 2: AB928/Etrumadenant + zimberelimab (mAb against PD-1) + docetaxel	Arcus Biosciences, Inc.	Prostate (advanced/incurable)	NCT04381832	1, 2 (R, OL)
A_2A_R/A_2B_R	AB928/Etrumadenant (dual antagonist) + zimberelimab (mAb against PD-1)	Arcus Biosciences, Inc.	Advanced malignancies	NCT03629756	1 (NR, OL)
A_2A_R/A_2B_R	AB928/Etrumadenant (dual antagonist) + Cisplatin/Radiation Therapy + Zimberelimab (mAb against PD-1)	Jennifer Choe in collaboration with Arcus Biosciences Inc	Head and neck	NCT04892875	1 (NR, OL)
A_2A_R/A_2B_R	Arm 1. AB928/Etrumadenant (dual antagonist) + zimberelimab (mAb against PD-1) + standard chemotherapeutic regime (mFOLFOX-6 + bevacizumab) Arm 2: AB928/Etrumadenant zimberelimab (mAb against PD-1) + AB680 (CD73 inhibitor)	Arcus Biosciences Inc.	Colon (metastatic)	NCT04660812	1, 2 (R, OL)
A_2A_R/A_2B_R	AB928/Etrumadenant (dual antagonist) + Carboplatin and Pemetrexed +/− Zimberelimab (mAb against PD-1)	Arcus Biosciences Inc.	Lung	NCT03846310	1 (NR, OL)
A_2A_R/A_2B_R	Arm A: AB928/Etrumadenant (dual antagonist) + Pegylated liposomal doxorubicin (PLD) Arm B: Etrumadenant + nanoparticle albumin-bound paclitaxel (NP) Arm C: Etrumadenant +PLD + IPI-549 (phosphoinositide-3-kinase-gamma inhibitor)	Arcus Biosciences Inc.	Breast (TN) or gynecologic	NCT03719326	1 (NR, OL)
A_2A_R/A_2B_R	AB928/Etrumadenant (dual antagonist) + mFOLFOX	Arcus Biosciences Inc.	Gastrointestinal	NCT03720678	1 (NR, OL)
A_2B_R	PBF-1129	Palobiofarma SL	NSCLC	NCT03274479	1 (NR, OL)
A_2A_R	Arm 1: AZD4635 + Durvalumab (mAb against PD-L1) Arm 2: AZD4635 + Oleclumab (mAb against CD73) Arm 3: AZD4635 + Durvalumab + Oleclumab	AstraZeneca	Prostate	NCT04089553	1, 2 (NR, OL)
A_2B_R	TT-4	Tarus Therapeutics	Gastrointestinal, hepatocellular, prostate	NCT04976660	1, 2 (OL)
A_2B_R	TT-4	Tarus Therapeutics	Gastrointestinal, hepatocellular, prostate	NCT04976660	1, 2 (OL)
A_2B_R	TT-4	Tarus Therapeutics	Gastrointestinal, hepatocellular, prostate	NCT04976660	1, 2 (OL)

* Only the target related to AR type is indicated. Abbreviations: AST: Advanced solid tumors; mAb or mab: monoclonal antibody; DB: Double blind; NA: Not applicable (safety assessment in healthy individuals); MC: multicenter; NHL: Non-Hodgkin lymphoma; NSCLC: Non-Small Cell Lung Cancer; NR: non-randomized; PC: Placebo-controlled; R: randomized; OL: Open label; TN: triple negative.

## Data Availability

Not applicable.
